# Genomic Tools and Animal Health

**DOI:** 10.3390/vetsci3030021

**Published:** 2016-09-07

**Authors:** Ricardo Zanella

**Affiliations:** School of Agronomy and Veterinary Medicine, College of Veterinary Medicine, University of Passo Fundo, Passo Fundo RS 99052, Brazil; ricardozanella@upf.br; Tel.: +55-54-3316-8485

**Keywords:** animal health, GWAS, genomic selection, infectious diseases

## Abstract

Animals have been selected to improve their productivity in order to increase the profitability to the producer. In this scenario, not much attention was given to health traits. As a consequence of that, selection was made for animals with higher production and a shortened productive life. In addition to that, the intense production system used in livestock has forced animals to be exposed to higher pathogen loads, therefore predisposing them to infections. Infectious diseases are known to be caused by micro-organisms that are able to infect and colonize the host, affecting their physiological functions and causing problems in their production and on animal welfare. Even with the best management practices, diseases are still the most important cause of economic losses in the animal industry. In this review article we have addressed the new tools that could be used to select animals to better cope with diseases and pathogens.

## 1. Introduction

Animal selection has historically been based on production traits, and not much attention was given to selection for health traits. This resulted in modern cows with high milk yields and reduced fertility, and shortened productive lives, caused by increased susceptibility to postpartum metabolic disorders. However, even in the selection for animals with higher production, it was possible to observe individual differences to challenges in the host response to diseases [1]. Selection has been used to control or manage infectious diseases, such as anthelmintics for parasite control, ascaricides for tick control and antibiotics to control bacterial diseases, and this have created major resistance problems. As a consequence of that, breeders and agricultural industries are now facing an intense pressure to select animals for enhanced disease resistance and tolerance with better welfare conditions [2].

In the last decades, important attention was given to acute infections caused by emerging diseases, especially those that infect humans from other host species. Those are called zoonotic diseases, and are caused by different pathogenic agents. The circulation of zoonotic agents between humans, animals, and the environment is a concern for animal and public health. According to the Institute of Medicine [3], more than 65% of emerging infectious diseases were caused by zoonotic agents, resulting in annually indirect economic losses of over $200 billion [4]. In this context it is expected that infectious disease will cause major economic losses in the livestock sector worldwide.

Many diseases will affect the animals’ productivity. Generally, it will occur in the early stages of infection (subclinical), when there is a lack of evidence of infection, which, if untreated, can progress to severe clinical disorders. The overall consequences of diseases on animal production are great. Generally, infected animals have lower performance rates, costs associated with animal treatment, increased mortality rates and possibly reproductive failure [5,6]. Complete knowledge of the disease mechanism is needed for the decision on the protocol and treatment to be used.

Vaccination and good management practices have been widely used to control and to avoid pathogens spreading in populations, providing variable levels of protection. However, the mechanism involving immune protection is not fully understood; therefore, not all the animals respond to the treatment, leading to the discovery of new approaches to address those failures [7,8]. It is common to observe in a vaccinated population some differences in the immune response between animals [7]. Also, differences in the severity of infection of animals exposed to similar doses of pathogens were found, suggesting a possible role of genetics involved with tolerance and resistance to infection [9]. Resistance can be defined as the host’s ability to resist an infection or to control the parasite lifecycle, and tolerance is the host’s ability to tolerate infection and show little or no effects of disease [9,10].

Up to this moment, the consequences of breeding animals for disease resistance or tolerance in relation to production traits are not known. It is known that selection for disease traits needs to be conducted within the herd, as opposed of production traits which can be done individually. This is because when working with infectious diseases, animals may infect each other directly or indirectly. Therefore, the health status between animals within the same herd is not independent of the other animals, since it depends on the host genetic makeup and also on the epidemiology of the disease.

Many factors have been identified to influence animal health; therefore, it is very complicated to evaluate and to study the host genetics and pathogen interaction. The most difficult concept in the experimental design is how to define and to establish the correct phenotype. How can we assume that an animal is resistant to a disease if we do not know the pathogen load that the animals have been exposed to or if the animal was even exposed? In this context, assumptions need to be made to evaluate animal exposure to pathogens, and it is expected that animals sharing the same environment are possibly experiencing the same amount and type of pathogens. The sensitivity and specificity of the diagnostic tools used are also very important to identify the epidemiologic status of animals in a herd. Different tests can result in a conflicting phenotypic classification, and therefore it can result in a broad range of outcomes with spurious results. Therefore, the success of genomic selection for health traits starts with correct phenotype collection and the accuracy of the diagnostic test. Animals can be infected without presenting clinical signs, or may be considered a false negative. The accuracy of the determination of the infectious status of the animal is directly related to the sensitivity and specificity of the diagnostic test [11], and different results can be obtained when different methodologies are applied. However, a true confirmation is obtained when the pathogen is isolated and sequenced from the organ or systems being affected by a specific disease. In addition to that, pathogen colonization will result in changes in the immune system of the animal, possibly altering the gene expression profile of tissues being infected or even having a global effect. Those changes in the host will have a direct or indirect effect on the disease phenotype.

In this review we have addressed and explored some of the challenges related to animal health, and some of the new tools that could be used to select animals to better cope with diseases and pathogens.

## 2. Infection-Disease Transmission

Several risk factors have been identified to influence whether an animal that was exposed to an agent will become infected or not. In addition to that, they can determine whether the infection will result in, sooner or later, clinical disease, depending on the host immune response. Some of those risk factors can be associated with animal and pathogen genetic composition, the nutritional state of the host at the time of infection, and the environmental conditions. Those factors can also be associated with the spread of the pathogen among other animals [1]. However, in all the cases of infection, the necessary condition is circulating levels of a specific pathogen in a population.

**Figure 1:**
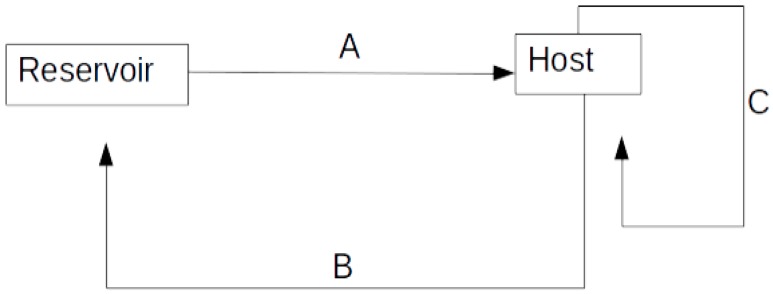
Model of disease transmission among or within populations.

The transmission of pathogens from a reservoir may be continuous or sporadic; however, once the infection is in the host population it may follow different pathways. In a population, the presence of a reservoir can directly transmit the pathogen to the host (Figure 1, Arrow A) in a sporadic or in a continuous way [1]. Once the host population becomes infected, it can be their own continuous source of infection (Figure 1, Arrow C), or result in a cross-contamination to the reservoir (Figure 1, Arrow B). The selection being imposed on the pathogens in the host may differ from the one being imposed in the reservoir; therefore, the genetic makeup in the pathogens on different populations may vary, resulting in different levels of disease severity and infectivity. The initial step in controlling the spread of diseases is the identification and control of those risk factors.

## 3. Selection for Resistance or Tolerance

To control the spread of diseases, we first need to control the infection (resistance), and second it is necessary to reduce the severity of the disease (tolerance). The main objective to control the infection is to reduce the number of infected animals (prevalence) in a herd or population, reducing the infectious pressure among the existing population. There are two basic genetic approaches that can be used to select animals to fight a pathogenic infection (resistance and tolerance).

**Figure 2:**
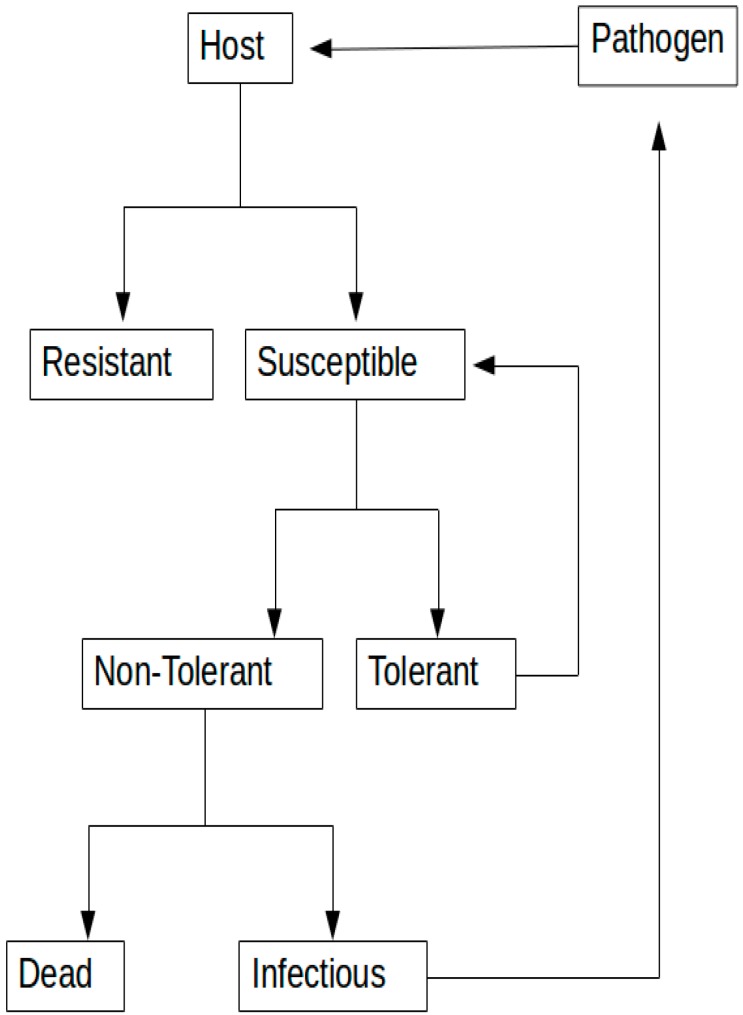
Schematic diagram of host × pathogen interaction-disease infection mode.

The first method is to select for animals that are resistant or less susceptible to infection; furthermore, susceptible animals can be subdivided as tolerant and less tolerant (Figure 2). Resistance was defined as the ability of an organism to prevent the infection when exposed to a pathogen [12]. Gonda and colleagues in 2007 [13] identified a QTL (Quantitative Trait Loci) on BTA20 affecting susceptibility to *Mycobacterium paratuberculosis* (*MAP*) infection in US Holstein cows. Using a similar approach for phenotypic classification, Settles and colleagues [14] tested animals for the presence of *MAP* with a different diagnostic method with better sensitivity and specificity. Using a genome-wide association analysis (GWAS), a chromosomal region on BTA3 associated with *MAP* tissue infection in a group of Holstein cows was identified. Investigation of this region on BTA3 using 42 single nucleotides to explore a 235 kb region identified a region of 10.6 kb, possibly harboring the causative mutation involved in *MAP* infection [15]. Further investigation using a gene set enrichment analysis identified the gene Endothelin-2 (*END2*) located near the SNP (Single Nucleotide Polymorphism) previously identified by Settles and colleagues [15] to be involved with *MAP* infection in cattle [16]. *EDN2* encodes a member of the protein family of secretory vasoconstrictive peptides and works as a ligand for the endothelin receptors, initiating an intracellular signaling cascade, which can modulate *MAP* tissue infection [9]. Therefore, this is a great positional and functional candidate gene involved in *MAP* infection.

To explore the role of *EDN2* and *MAP* infection, nuclear extracts from freshly harvested ileo-cecal lymph nodes from Holstein cows were used to characterize differences in the binding affinities of the sequences harboring the significant SNPs on BTA3. Luciferase reporter assays were performed to confirm the transcriptional activity of *EDN2*, demonstrating mobility shifts, indicating that one or more variants in the promoter of *EDN2* may result in susceptibility to *MAP* tissue infection in Holstein cattle [17].

**Figure 3:**
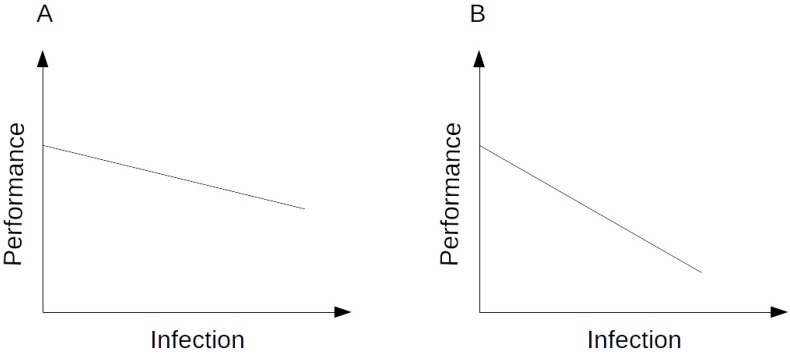
Susceptible animals can be tolerant or non-tolerant; a higher infection load associated with lower losses in the performance is an indication of higher tolerance (**A**) where increased losses in performance caused by increased infection load is an indication of a less tolerant animal (**B**).

The second approach that can be used is to select for animals which are genetically tolerant to the pathogenic infection. Raberg and colleagues [12] presented empirical evidence for genetic variation leading to tolerance in animals using a mouse model to study malaria. Their definition of tolerance was based on individuals who respond to a pathogenic infection in such a way that limits the damage (fitness) to the host without affecting the pathogen burden. Tolerance is generally described as the relationship between the levels of infection intensity and fitness. Therefore, in a tolerant animal, the amount of infection has little or no effect on the fitness of the individual [10,15]. Production performance tends to be reduced in animals with higher pathogen loads, and this is illustrated in Figure 3, which shows a representation of the performance and level of infection.

Genetic tolerance has been evaluated in cattle that were infected with *MAP* and its genetic association was tested using a GWAS, and a region on BTA15 encoding the gene *GNA12* was associated. For this, tolerance was measured as the amount of *MAP* fecal shedding relative to the levels of *MAP* tissue infection [10,15]. High levels of fecal shedding on Johne’s positive animals are generally associated with disease severity, since they are negatively correlated with milk production, fat content and protein content, lower productive life and an increased number of days between pregnancies, as well as high disease transmission rates to the herd [13,18,19,20]. Therefore, levels of *MAP* fecal shedding could be used as an accurate measurement of animal fitness.

Differences in the genetic background between animals also need to be taken into account when evaluating association with genetic markers, to reduce the possibility of identifying erroneous markers involved with the phenotype being studied. This test can be conducted using the pair-wise identical by state test (IBS) computed between all the animals in the study and plotted using a multi-dimensional scaling plot MDS for better visualization.

## 4. Selection for Better Immune Response

Another approach that could be used is to select for animals that will have a better response when challenged with pathogens. Vaccination is considered the most powerful tool used to control and to diminish the parasite burden, reducing the clinical signs and the transmission of the pathogen within animals [21]. However, animal response to vaccination can vary within individuals, with some individuals not responding at all or having different levels of protection [22].

In the livestock sector, most of the economic losses have been associated with infection with pathogens. To overcome those losses, several strategies have been proposed and implemented with unsatisfactory results. For example, influenza virus is still considered the most prevalent respiratory pathogen circulating among animals and humans, causing a major impact to the economy and the population’s health. Therefore, there is a need to develop new methodologies to increase the efficacy of the current methods.

Here, we present results of an association study of genetic markers with the immune response to influenza vaccination in a swine herd [7]. Animals were vaccinated with an inactivated H1N1pdm virus and tested for the association of genetic markers with immune response efficacy to influenza vaccination (IAV). The animal immune response was measured based on the presence or absence of antibodies to IAV (qualitative measurement), and also based on the levels of antibodies produced by the animals (quantitative).

Using the response to vaccination as a qualitative phenotype, a region on *SSC12* was identified to be associated with the immune response. When we tested the efficacy of the response to the vaccination as a quantitative phenotype, four additional associated chromosomes were identified: *SSC0*, *SSC1*, *SSC7* and *SSC15* [7]. These results suggest the complexity of the immune traits when used to test their interaction with host genetics.

**Figure 4:**
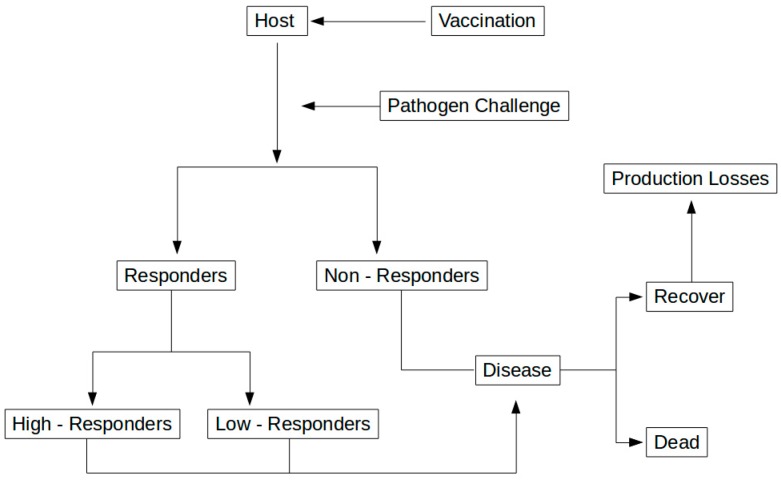
Representation of animal response to vaccination.

However, even in vaccinated animals we can have responders that will become infected, and those animals can recover from infection but will present losses in production. Those losses are generally associated with lower production or even reproductivity failure (Figure 4).

## 5. New Approaches to Old Problems

The great advantage of high-throughput sequencing is the ability to generate a large amount of genomic information to address complex problems. In addition to that, the use of genomic sequencing for the identification of specific pathogens associated with diseases has been facilitated, allowing for the identification of genomic variations associated with the pathogenicity of specific agents and the sensitivity to specific treatment drugs.

In dairy cows, clinical mastitis is considered a complex health disorder which will impact the farm’s profitability and the animal welfare. The heritability associated with this disorder can range from 0.01 to 0.42, indicating the involvement of genetic components with the appearance of this disorder [23]. Using whole genomic association studies (GWAS), genetic markers were identified on chromosomes 2, 14 and 20 as associated with susceptibility to this disorder, indicating a polygenic effect [23]. Some of the genes located near the associated markers were involved with immune response activation and mammary gland metabolism. Despite those findings, it was proposed that susceptibility to clinical mastitis is regulated by different genes related to immune response which are pathogen-specific; therefore, animal selection for resistance to a specific pathogen might not work for different pathogens.

Recent studies have applied the RNA sequencing technique to unsolved biological problems, especially for the identification of genes involved with illness [24,25,26]. This new methodology uses the whole transcriptome sequencing of a specific tissue at a specific time point. This might be a useful tool to investigate the genetic mechanisms involved with the host infection and with the host immune response to an infection. Beside the advantage of using a small number of animals, this methodology will possibly be able to give the whole picture of what is happening transcriptomically when the animal is facing a challenge, especially within an infection.

The host-pathogen interaction has been described in a study using the RNA-Seq approach, which investigated both viral and host gene expression in a mouse model after H1N1 infection. An important gene involved with viral replication and host defense has been identified in this model. The use of this methodology could be applied to elucidate the host-pathogen interactions, increasing the knowledge of genes involved with host susceptibility or survival during infections [27].

As an example, animals with different genotypes (AA, AG, GG) for a specific marker on exon 5 of *EDN2* (the gene involved with resistance to *MAP* infection) had different levels of its expression in the lymph nodes. This indicates a possible suppression of the gene expression in animals with a specific genotype when exposed to specific challenges [28]. However, this study was conducted evaluating the transcription profile using only one gene in one tissue, without accounting for the effect of other genes. The use of high-throughput sequencing offers us an opportunity for the identification of global transcriptomic changes during specific challenges in different tissues at the same time.

## 6. Conclusions

Genetic selection for animals that are resistant, or tolerant or for animals that have a better immune response to vaccination can help diminish the impact of diseases in the livestock sector. However, it is not known what the best strategy is and what the long-term consequences are when selecting for those traits. However, if selection for those traits can reduce human exposure to infectious pathogens, it offers a potential to reduce the health risks in a population.
